# 磷酸化肽异构体的分子印迹识别最新研究亮点

**DOI:** 10.3724/SP.J.1123.2026.08007

**Published:** 2026-09-08

**Authors:** Luxi CHEN, Lianghai HU

**Affiliations:** 吉林大学超分子化学生物学研究中心，超分子结构与材料全国重点实验室，吉林大学生命科学学院，吉林 长春 130012

蛋白质磷酸化是细胞信号调控过程中重要的翻译后修饰形式，参与细胞增殖、免疫响应以及代谢调节等多种生命过程。异常磷酸化事件与癌症等多种疾病的发生发展密切相关。随着高分辨质谱技术的发展，磷酸化蛋白质组学已成为解析蛋白质功能调控的重要研究手段。然而，在复杂生物样品中，磷酸化蛋白通常具有丰度低、动态变化快等特点，不同修饰位点形成的位置异构体具有相同的前体离子质量、相近的色谱保留行为和高度重叠的碎片离子，因而难以准确区分。TiO₂和固定化金属离子亲和色谱（immobilized metal ion affinity chromatography， IMAC）等方法主要依据磷酸基团与材料之间的配位作用实现广谱富集，难以进一步识别磷酸基团所处的局部序列；磷酸化抗体虽可提高靶向性，但对相邻位点或多重磷酸化肽可能存在交叉识别，因而仍难以实现磷酸化位置异构体的准确区分。

近日，瑞典马尔默大学Börje Sellergren教授与南丹麦大学Ole N. Jensen教授等^［1］^在*Nature Chemical Biology*发表研究论文，报道了一种序列选择性的磷酸化肽富集策略。如图1所示，该研究构建了一种对磷酸化位点具有特异识别功能的分子印迹聚合物（molecularly imprinted polymer， MIP），结合液相色谱-高分辨质谱联用技术，实现了复杂细胞样本中蛋白质磷酸化位点异构体的精准识别与分析。

**图1:**
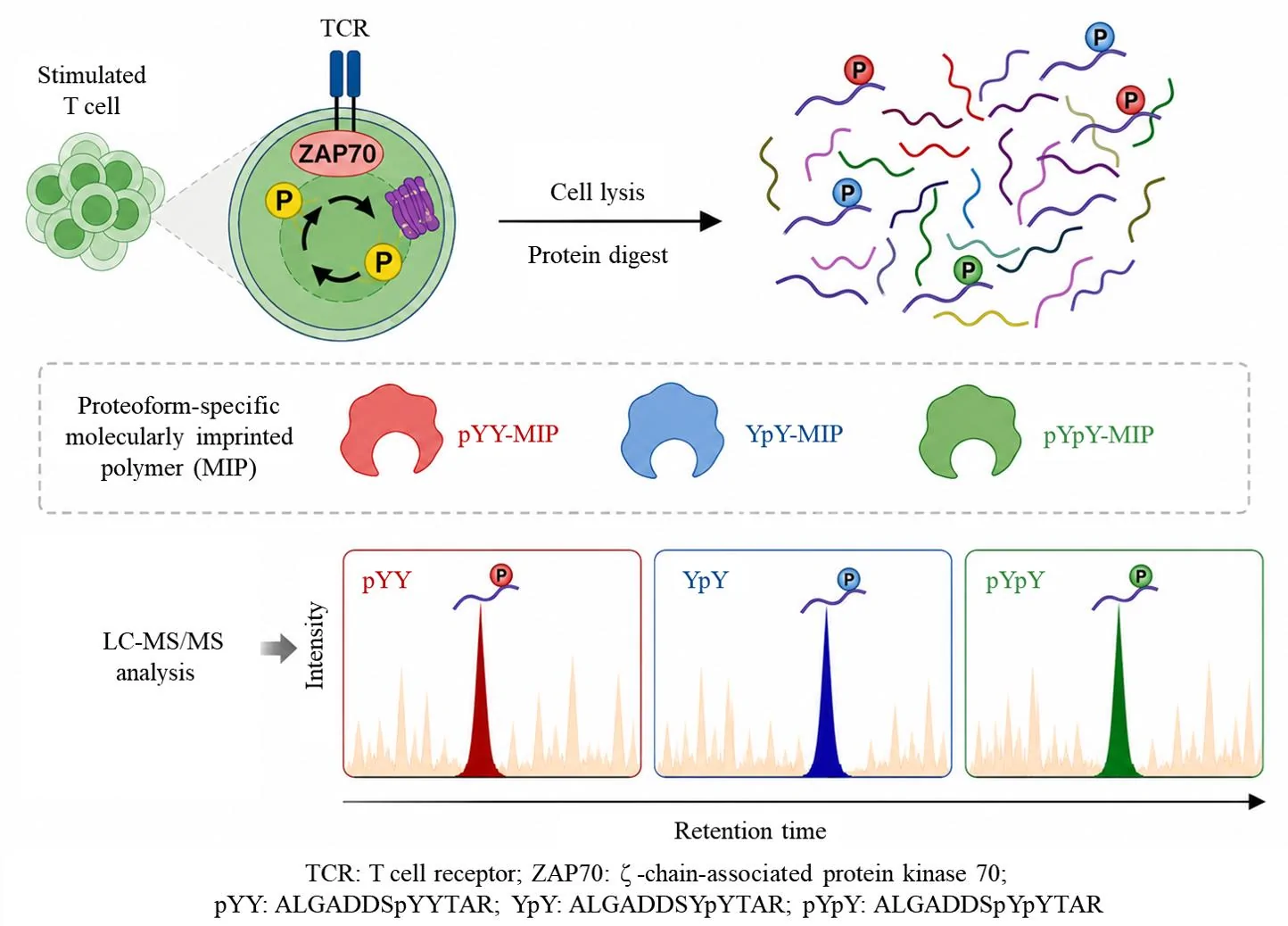
序列选择性MIP与LC-MS/MS联用对磷酸化肽异构体的识别与分析

ζ链相关蛋白激酶70（zeta-chain-associated protein kinase 70， ZAP70）作为T细胞受体信号通路中的关键激酶，其结构域中酪氨酸492（Y492）和酪氨酸493（Y493）位点的磷酸化状态与蛋白质活性调节密切相关。然而，由于两个位点在氨基酸序列中相邻，不同磷酸化形式产生的肽段具有高度相似的结构特征，传统磷酸化肽富集方法难以准确区分。针对这一问题，该文献根据不同磷酸化模式构建了3类具有序列选择性的分子印迹聚合物，包括pYY-MIP、YpY-MIP和pYpY-MIP，分别用于识别不同单磷酸化肽段pYY（Y492磷酸化）、YpY（Y493磷酸化）以及双磷酸化肽段pYpY（Y492和Y493同时磷酸化），并以非磷酸化肽段YY为模板制备YY-MIP作为对照。通过在聚合过程中引入目标磷酸化肽模板，形成与目标序列、空间结构及修饰状态相匹配的识别空腔，从而赋予聚合物对特定磷酸化异构体的选择性结合能力。竞争结合与等温吸附实验表明，不同MIP材料均表现出对目标磷酸化肽的优先识别能力。目标肽段与对应MIP之间的最大结合容量达到2.5~3 μmol/g，而非互补肽段仅为0.5~1 μmol/g；解离常数处于微摩尔浓度范围，能够有效区分结构高度相似的磷酸化肽异构体。在复杂蛋白质背景中，pYY-MIP、YpY-MIP和pYpY-MIP分别实现了对应pYY、YpY和pYpY肽段的选择性富集。当目标肽段与背景肽段物质的量比为1∶10时，各类材料均表现出较好的选择性；即使在更低丰度条件（1∶100和1∶1 000）下，仍可实现对目标磷酸化肽的富集，显示出其在复杂样品分析中的应用潜力。

为验证该方法在真实生物样本中的应用价值，该文献利用Jurkat T细胞建立信号刺激模型，对ZAP70不同磷酸化状态进行分析。CD3（cluster of differentiation 3）抗体刺激可增强Y493位点的磷酸化，而联合H₂O₂处理可促进Y492位点的磷酸化。经凝胶分离、胶内酶解后，利用不同分子印迹聚合物进行选择性富集，并结合LC-MS/MS分析，可检测到ZAP70 Y492/Y493位点对应的不同磷酸化形式，包括非磷酸化肽YY、两种单磷酸化异构体pYY和YpY以及双磷酸化肽pYpY。值得注意的是，传统pY492抗体虽然能够识别Y492磷酸化肽段pYY，但对同时含有Y492和Y493磷酸化的双磷酸化肽pYpY存在一定交叉反应，限制了其对相邻磷酸化位点异构体的精确区分。进一步分析表明，所有通过pYY-MIP、YpY-MIP和pYpY-MIP富集获得的含磷酸酪氨酸肽段均来源于ZAP70，表明该策略能够在复杂细胞蛋白质背景中实现目标磷酸化位点的选择性捕获。此外，不同聚合物在实际样品分析中表现出一定的差异：YpY-MIP具有较好的综合富集能力，可以高选择性捕获单磷酸化目标肽；pYY-MIP更倾向于富集双磷酸化相关肽段；而pYpY-MIP则由于可能优先结合含多个修饰位点的较长肽段，对目标肽段回收能力有限。尽管不同MIP的富集效率尚存在不均一性，但鉴于其对磷酸化肽的特异识别能力，此类MIP可直接应用于全细胞裂解液中目标磷酸化肽的选择性富集，兼具优异的化学与生物稳定性，且与蛋白质组学分析流程良好兼容。

磷酸化分子印迹研究多以磷酸基团或磷酸化氨基酸侧链为识别对象，虽已用于酪氨酸、组氨酸等修饰肽段的富集，但对序列异构体的区分未见报道。本研究将分子印迹材料的识别维度由磷酸基团的广谱亲和拓展至磷酸化特异序列的精准识别，并结合LC-MS/MS的定性定量分析，实现了相邻磷酸化位点对应肽段的选择性富集与分析。该分子印迹受体具有结构可设计性与高生物稳定性等特点，为复杂生物样品中蛋白质翻译后修饰的分析提供了新的研究范式。
